# Supplementary material for: Complex‐centric proteome profiling by SEC‐SWATH‐MS
Source: Mol Syst Biol. 2019 Jan 14;15(1):e8438. doi: 10.15252/msb.20188438 (PMC6346213; doi:10.15252/msb.20188438)
Supplement: Supplementary file 8 — Dataset EV7 [file MSB-15-e8438-s008.zip › feature_plots_string/Q05086.pdf]

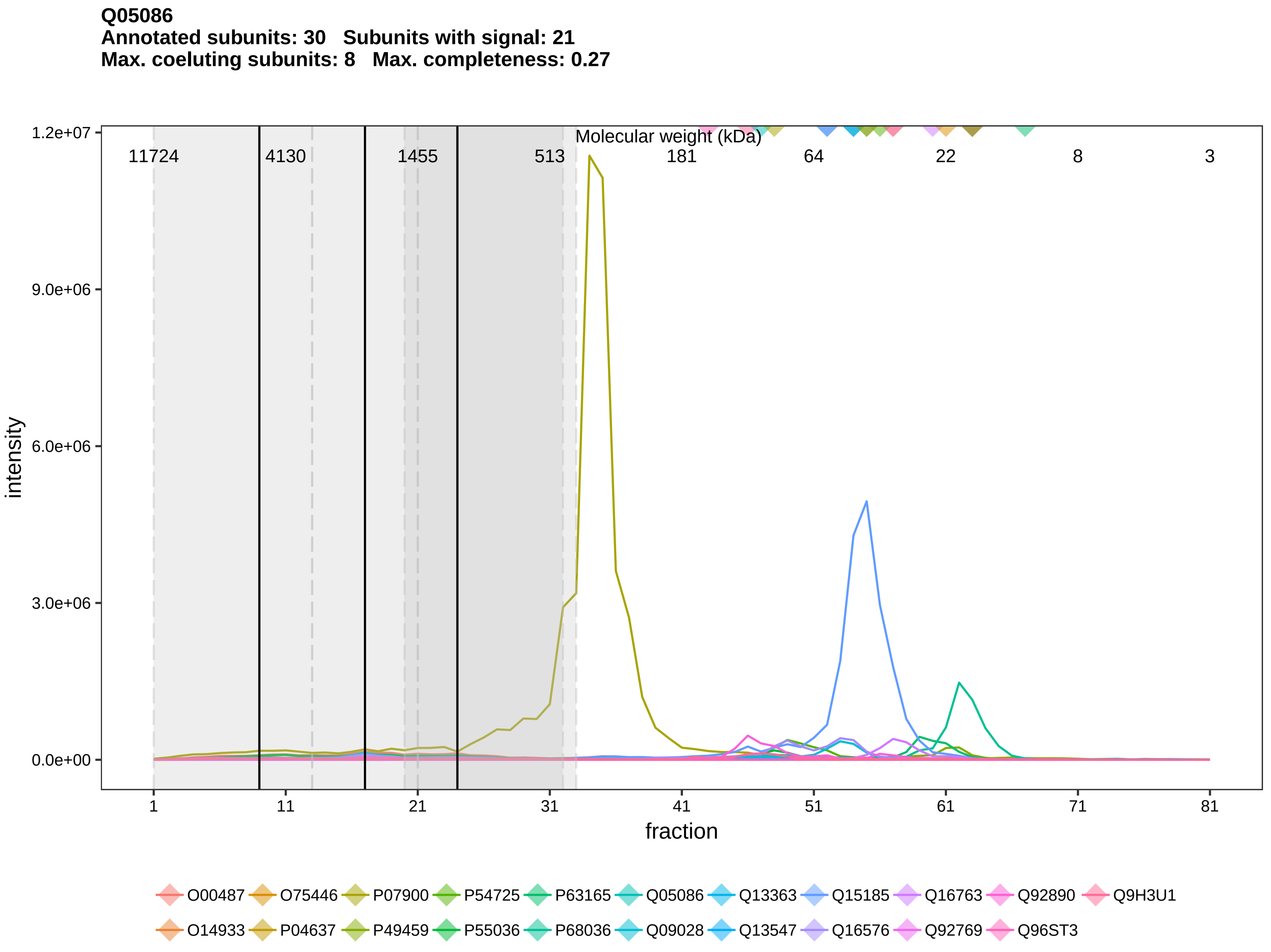

Q05086
Annotated subunits: 30   Subunits with signal: 21
Max. coeluting subunits: 8   Max. completeness: 0.27
Molecular weight (kDa)
11724
4130
1455
513
181
64
22
8
3
intensity
1.2e+07
9.0e+06
6.0e+06
3.0e+06
0.0e+00
fraction
1
11
21
31
41
51
61
71
81
O00487
O75446
P07900
P54725
P63165
Q05086
Q13363
Q15185
Q16763
Q92890
Q9H3U1
O14933
P04637
P49459
P55036
P68036
Q09028
Q13547
Q16576
Q92769
Q96ST3
